# Making Open Science the Default: Creating Lab Practices to Promote Transparency

**DOI:** 10.1093/geroni/igaa057.1881

**Published:** 2020-12-16

**Authors:** Kendra Seaman

**Affiliations:** The University of Texas at Dallas, Dallas, Texas, United States

## Abstract

Many factors disincentivize researchers, particularly junior faculty members, from implementing open science practices. One way to make open science less burdensome is integrate open science methods with existing procedures. I will describe my ongoing efforts to establish open science practices as the default in my laboratory. These strategies include (1) creating and updating a lab manual to set expectations for openness, (2) articulating a standard operating procedure for creating, preregistering, and managing a new project, (3) establishing clear organizational structures for data, code, and data products, and (4) training lab members on the use of these and other open science tools like GitHub. These strategies provide a model for both junior researchers starting a lab and more established researchers who want to build transparency into their research practices. Ultimately, implementing open science methods will improve lab workflows and improves the overall quality of our science.

